# Degradation of Sodium Acetate by Catalytic Ozonation Coupled with MnOx/NiOOH-Modified Fly Ash

**DOI:** 10.3390/toxics12060412

**Published:** 2024-06-04

**Authors:** Ruifu Chen, Hao Zhang, Shengyu Shao, Huajun Xu, Kaicheng Zhou, Yinzhi Jiang, Pengfei Sun

**Affiliations:** Key Laboratory of Surface & Interface Science of Polymer Materials of Zhejiang Province, School of Chemistry and Chemical Engineering, Zhejiang Sci-Tech University, 928 Second Avenue, Xiasha Higher Education Zone, Hangzhou 310018, China

**Keywords:** fly ash, manganese oxide, nickel oxide hydroxide, ozone catalysis, sodium acetate

## Abstract

Fly ash, a type of solid waste generated in power plants, can be utilized as a catalyst carrier to enhance its value-added potential. Common methods often involve using a large amount of alkali for preprocessing, resulting in stable quartz and mullite forming silicate dissolution. This leads to an increased specific surface area and pore structure. In this study, we produced a catalyst composed of MnO_x_/NiOOH supported on fly ash by directly employing nickel hydroxide and potassium permanganate to generate metal active sites over the fly ash surface while simultaneously creating a larger specific surface area and pore structure. The ozone catalytic oxidation performance of this catalyst was evaluated using sodium acetate as the target organic matter. The experimental results demonstrated that an optimal removal efficiency of 57.5% for sodium acetate was achieved, surpassing even that of MnO_x_/NiOOH supported catalyst by using γ-Al_2_O_3_. After loading of MnO_x_/NiOOH, an oxygen vacancy is formed on the surface of fly ash, which plays an indirect oxidation effect on sodium acetate due to the transformation of ozone to •O_2_^−^ and •OH over this oxygen vacancy. The reaction process parameters, including varying concentrations of ozone, sodium acetate, and catalyst dosage, as well as pH value and the quantitative analysis of formed free radicals, were examined in detail. This work demonstrated that fly ash could be used as a viable catalytic material for wastewater treatment and provided a new solution to the added value of fly ash.

## 1. Introduction

Fly ash is a kind of solid waste produced by coal-fired power plants, the main components of which are Al_2_O_3_ and SiO_2_, and also contains a small amount of metal oxides such as CaO, Fe_2_O_3_, and MgO [1,2]. It is a kind of industrial waste that is highly polluting to the environment and brings many troubles to daily life [3]. Therefore, the treatment and utilization of fly ash have become urgent. However, due to its excellent thermal stability, fly ash has been increasingly used in adsorption and catalytic materials in recent years [4]. Since the pristine fly ash is composed of passivated mullite and quartz, its specific surface area is particularly small. It has been reported in the literature that a simple acid-base treatment by one-step (HNO_3_ or NaOH) or two-step (NaOH/HNO_3_ or HNO_3_/NaOH) leaching partial dissolution, which significantly improved the physicochemical properties of the inefficient fly ash [5]. Therefore, fly ash is also a very potential catalyst carrier. For example, Zn-Cr layered double oxide/fly ash (ZnCrLDO/FA) composites were synthesized as a photocatalyst by an in situ co-precipitation and calcination process, which exhibited significant environmental antibiotic degradation performance (10 mg/L of ciprofloxacin with 98% conversion within 120 min) [6]. The fly ash-coated ceramic cobalt ferrite (CoFe_2_O_4_) nanocomposite photocatalysts (CFA-CoFe_2_O_4_) were also synthesized using a hydrothermal method, resulting in the degradation of 10 ppm methylene blue with 99% efficiency within a reaction time of 60 min [7]. Furthermore, investigations have also been conducted to synthesize zeolites and mesoporous molecular sieves (e.g., MCM-41, SBA-15, and ZSM-5) as precursors for catalytic materials utilizing silica and aluminum sources derived from fly ash [8,9,10]. The synthesis of SBA-15 mesoporous molecular sieves was achieved by utilizing fly ash as a silica source, while CoMn catalysts were loaded onto it as a carrier and activated with peroxomonosulfate (PMS) for the degradation of rhodamine B (RhB) in aqueous solution [11].

The increasing presence of recalcitrant toxic organic pollutants has propelled advanced oxidation processes (AOPs) into the forefront of current research, thereby imposing higher demands on catalyst preparation in terms of raw material cost and synthesis process [12]. Catalytic ozone oxidation is widely regarded as a superior method among various AOP techniques because of its wide adaptability to pollutant degradation, mild reaction conditions, and thorough degradation [13]. Studies have shown that metal oxides (e.g., Fe_2_O_3_, MnO_x_, and NiO) loaded on mesoporous materials (silica, alumina, zeolite) are effective catalytic materials for ozone oxidation [14,15,16]. For example, the degradation of 2,4-dichlorophenoxyacetic acid catalyzed by Ni/TiO_2_ [17] and the degradation of oxalic acid catalyzed by alumina-loaded nickel oxide [15]. Both of these catalysts enable the activation of ozone in solution and the generation of free radicals through their active sites on the catalyst surface, resulting in rapid oxidation removal of organic matter. However, these supported oxide catalysts necessitate carrier screening and oxide calcination processes in the synthesis method, posing challenges to energy consumption and material costs in mass production. Alumina is the most widely used carrier for synthesizing ozone catalysts; the large usage and high unit price are the key factors affecting the cost of these supported ozone catalysts [18]. Obviously, the utilization of fly ash as a carrier for industrial catalysts, coupled with the elimination of the energy-intensive calcination process via a simplified chemical reaction synthesis method on its surface, paves the way for an innovative and energy-efficient approach to catalyst production in pollution control industries.

Due to their high density of hydroxyl sites, numerous surface-active sites, fast electron transfer rates, and metallic hydroxyl oxides are increasingly being used in water treatment research. For instance, in PMS systems, NiOOH degrades sulfadiazine contaminants completely within 90 min at a rate 5.3 times higher than that of Ni_2_O_3_, 2.5 times higher than that of NiO, and 2.2 times higher than that of Ni(OH)_2_ [19]. MnOOH was also used as an active agent to degrade p-chloroaniline wastewater in the PMS system. The hydroxyl group on the surface of MnOOH acts as a bridge, connecting PMS and the catalyst and activating PMS molecules as the active substance in the reaction to degrade p-chloroaniline [20]. The in situ generation of oxygen-rich vacancies (Fe_0.67_Mn_0.33_)OOH catalyst primarily enhances electron transfer at the interface to promote PMS activation, while the existence of surface hydroxyl groups enhances the generation of hydroxyl radicals [21]. In the process of ozone catalytic oxidation, the presence of numerous hydroxyl groups on the surface of hydroxyl oxide facilitates the adsorption of ozone onto the catalyst surface and enhances the activation of ozone molecules into active oxygen species. As a result, this leads to an expedited degradation of organic pollutants. For example, synthetic α-FeOOH degraded nitrobenzene wastewater under pH = 6.7 and low concentration of ozone conditions, resulting in a 65% degradation of nitrobenzene within 20 min, which is twice as efficient as degradation by ozone oxidation alone [22]. The synthesis of Co-FeOOH nanocrystals facilitated the ozonation process for atenolol in water. The hydroxyl group on the surface promotes the adsorption and decomposition of ozone to form •OH, significantly improving atenolol degradation during catalytic ozonation [23], and the total organic carbon (TOC) is effectively reduced. MnOOH nanorods were also used to inhibit bromate formation in wastewater containing organic pollutants. Compared with the single ozonation process, MnOOH nanorods achieved an inhibition rate of up to 54.1%. On MnOOH’s surface, Mn(IV) accepts electrons forming transient Mn(II)/Mn(III) lattice oxygen species along with •O_2_^−^, where Mn(II)/Mn(III) facilitates bromate reduction to bromide during catalytic ozone oxidation [24]. Therefore, metal hydroxyl oxide can serve as an effective catalyst for the ozone oxidation process, facilitating the degradation of organic pollutants. Moreover, the synthesis of metal hydroxyl oxides bypasses the high-temperature calcination process required for synthesizing metal oxides, which creates new opportunities for simple, environmentally friendly, and low-cost methods to degrade organic pollutants.

Therefore, in this work, the catalytic ozonation process was investigated using catalysts containing Ni_y_Mn_1-y_OOH, which were prepared by utilizing pretreated fly ash as a carrier. Nickel and manganese are among the most abundant elements on Earth and are less toxic than other transition metals. NiOOH and Mn_2_O_3_ are produced via a one-step reaction of Ni(OH)_2_ and KMnO_4_, eliminating the need for high-temperature calcination of synthetic metal oxides, which makes the reaction more environmentally friendly. Additionally, Ni^3+^ in NiOOH can be converted to Ni^2+^ and Ni^4+^ to enhance ozone oxidation. While ozone can directly degrade most organic matter, acetic acid, as one of the common terminal intermediates in the oxidative degradation process of organic pollutants by ozone, is resistant to ozone and difficult to mineralize. This severely hampers the effectiveness of wastewater treatment [25]. Therefore, we chose sodium acetate as the target pollutant for catalytic ozonation. The synthesized catalyst was characterized using XRD, SEM, TEM, BET, ICP, XPS, and EPR. The catalytic activity of x-Ni_y_Mn_1−y_OOH/ACFA for the ozonation of sodium acetate was investigated to examine the effects of reaction conditions such as ozone concentration, catalyst dosage, sodium acetate concentration, and initial pH on the catalytic ozonation. We also identified reactive oxygen species by EPR characterization and quenching experiments and quantified reactive oxygen generated by the reaction by PL and NBT methods to explore its catalytic reaction mechanism.

## 2. Materials and Methods

### 2.1. Synthesis of Catalysts

**Figure 1:**
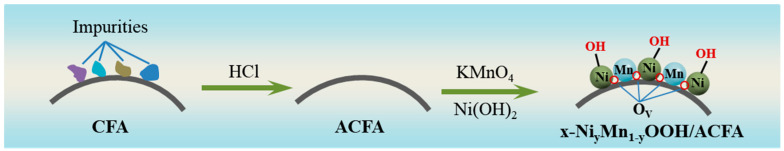
Preparation process of fly ash catalyst.

The fly ash used as the raw material was sourced from China Energy Group’s Zhoushan Power Plant. The schematic diagram of catalyst synthesis is shown in Figure 1. The fly ash was subjected to a pretreatment procedure as follows: 10 g of fly ash was combined with 100 mL of HCl solution (1.2 mol/L), and the mixture was heated and stirred at a temperature of 85 degrees Celsius for a duration of 2 h. Subsequently, it underwent rinsing with deionized water until reaching neutrality, followed by drying at a temperature of 60 degrees Celsius. The resulting sample obtained from this process was designated as ACFA.

The acidified CFA was used as the carrier for Ni-Mn loading, and the synthetic catalyst was named x-Ni_y_Mn_1−y_OOH/ACFA, where x represents the wt.% of Ni-Mn atoms on the surface of ACFA, and y/1-y represents the different Ni/Mn ratios. Taking the synthesis of 0.2-Ni_0.67_Mn_0.33_OOH/ACFA catalyst as an example, 10 g of ACFA was added to 50 mL of 5 mol/L KOH solution with constant stirring. Then, 6.345 g of Ni(OH)_2_ and 5.745 g of KMnO_4_ solution were added, and the mixture was stirred at 90 °C for 15 h. Then, it was centrifuged to neutral by washing with deionized water and dried at 60 °C. Finally, several samples were synthesized with the ratio of Ni/Mn at 1:5, 4:2, and 5:1 and the wt.% of Ni-Mn atoms at 8%, 20%, and 30%, respectively.

### 2.2. Characterization

The X-ray powder diffractometer (Haoyuan DX-2700, Dandong, Liaoning, China) equipped with Cu Ka radiation (35 kV, 25 mA) was utilized to measure the wide-angle X-ray diffraction (XRD) patterns of various ACFA samples. The scanning electron microscope (SEM, Hitachi SU-8100, Chiyoda, Japan) was utilized to observe and document the morphology. The microstructure of the x-Ni_y_Mn_1−y_OOH/ACFA surface was further examined using a transmission electron microscope (TEM, JEM 2100, Tokyo, Japan). The ASAP 2020 analyzer from Micromeritics, Norcross, GA, USA, was utilized to measure the nitrogen adsorption–desorption isotherms. The specific surface area and pore size distribution were determined using the BET and BJH methods developed by Brunauer–Emmett–Teller and Barrett–Joyner–Halenda, respectively. The iCAP PRO XP ICP-OES emission spectrometer from Thermo, Waltham, MA, USA, was utilized to obtain the Inductively Coupled Plasma Emission Spectra (ICP-OES) measurements. The ICP elemental determination was carried out directly after sufficient acid digestion of 0.1 mg of the sample. The X-ray photoelectron spectroscopy (XPS) analysis was conducted on a Kratos AXIS Ultra DLD spectrometer (Shimadzu, Kyoto, Japan) with Al Ka *X*-rays as the excitation source. The measurement of electron paramagnetic resonance spectroscopy (EPR) was conducted using an A300 EPR spectrometer manufactured by Bruker in Germany.

### 2.3. Catalytic Ozonation Reaction

**Figure 2:**
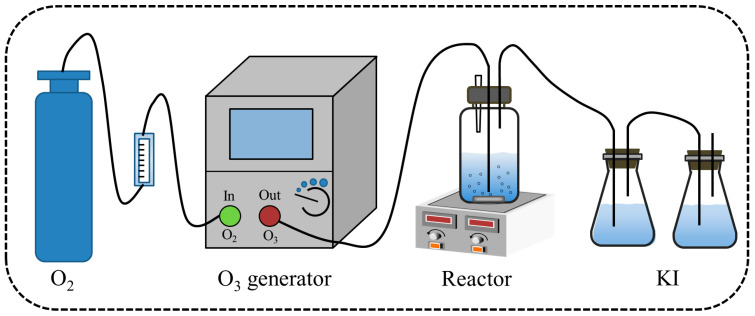
Diagram of a device for catalytic ozone oxidation reaction.

The catalytic ozone reaction was conducted in a self-designed reactor using a semi-intermittent mode, as depicted in Figure 2. In this process, 600 mL of sodium acetate solution (initial pH 7.5, sodium acetate concentration ranging from 50 to 200 mg/L) was added to the reactor, along with 1~5 g/L of catalyst in the reaction solution. The initial pH of the reaction was adjusted by adding either a 0.1 mol/L NaOH or HCl solution. During the ozonization experiments, ozone was continuously generated by passing high-purity oxygen from an oxygen cylinder into an ozone generator (M-1000, Tonglin, Beijing, China). The resulting ozone/oxygen gas mixture was then bubbled into the reactor at a flow rate of 200 mL/min with an ozone concentration of 50 mg/L. Any excess ozone produced during this process was absorbed using a KI solution. For the catalytic ozonation experiments, samples measuring 4.0 mL were taken at specific time intervals and filtered through a 0.45 µm inorganic membrane to remove any impurities present before quenching any remaining residual ozone by adding a 0.01 mol/L Na_2_S_2_O_3_ solution. Finally, the sodium acetate removal efficiency was assessed by employing a COD meter (COD-571, Leici, Shanghai, China) to measure chemical oxygen demand.

### 2.4. Reactive Species Detection

Nitroblue tetrazolium (NBT) has the ability to undergo a reaction with •O_2_^−^, and its concentration can be determined using ultraviolet spectrophotometry as an indirect method for assessing the generation of •O_2_^−^. A catalyst of 0.2-Ni_0.67_Mn_0.33_OOH/ACFA was introduced into the NBT solution with a concentration of 4.2 × 10^−5^ mol/L. To achieve adsorption equilibrium, oxygen was introduced and stirred for a duration of 60 min. After that, the reaction was initiated by introducing ozone. At specific time intervals, 4.0 mL of the reaction solution was measured and filtered through a 0.45 µm inorganic filtration membrane. The change in intensity at 259 nm was recorded using a UV-visible spectrophotometer (UV-2600, Shimadzu, Japan).

Terephthalic acid (TA) undergoes a reaction with •OH resulting in the formation of 2-hydroxyterephthalic acid (TAOH) exhibiting significant fluorescence intensity. The fluorescence spectrophotometry technique was employed to detect the production of TAOH with an excitation wavelength set at 315 nm. Additionally, a subsequent analysis was conducted to quantify the concentration of •OH generated in the solution during the catalytic reaction. The process involved dispersing the catalyst in a solution of 2.0 mmol/L NaOH and 2.0 mmol/L TA, followed by achieving adsorption equilibrium via stirring for an hour while passing oxygen. Then, ozone was introduced, and at specific time intervals, a measurement of 4.0 mL of the reaction solution was taken and filtered through a 0.45 µm inorganic filter membrane. Fluorescence intensity measurements were conducted using a fluorescence spectrophotometer (Horiba FluoroMax-4, Horiba, Kyoto, Japan). Fluorescence conditions included an excitation wavelength of 315 nm with a slit width of 2 nm.

## 3. Results and Discussion

### 3.1. Catalytic Ozonation Performance of x-Ni_y_Mn_1−y_OOH/ACFA

**Figure 3:**
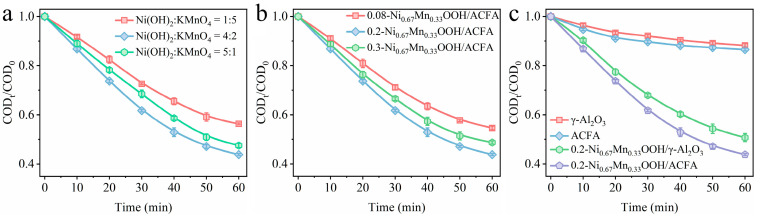
(**a**) The degradation performance of sodium acetate over samples with different ratios of Ni/Mn; (**b**) the degradation performance of sodium acetate over samples with different loading amounts of metal species; (**c**) the degradation performance of sodium acetate over samples with different catalyst carriers; (ozone concentration: 50 mg/L, catalyst dosage: 3 g/L, sodium acetate concentration: 150 mg/L, pH: 7.5, and temperature: 25 °C).

Sodium acetate is the end product of the organic oxidation process, so using sodium acetate as the target degradation material in the ozone catalytic oxidation process can more directly reflect the practical use value of the catalyst. As shown in Figure 3a, the degradation rate of sodium acetate during catalytic ozonation was investigated by varying the loading amounts of Ni and Mn at 20 wt.%. The degradation rate of sodium acetate was observed to exhibit a remarkable enhancement, soaring from 43.7% to an impressive 56.2%, concomitant with the augmentation in Ni/Mn ratio from 1:5 to 4:2. When the ratio of Ni(OH)_2_ to KMnO_4_ arrives to 5:1, the sodium acetate degradation rate decreased to 52.5%. Therefore, the surface Ni/Mn ratio has a significant impact on the degradation of sodium acetate, primarily due to its influence on the formation of NiOOH or MnO_x_ during surface reactions. The direct oxidation of sodium acetate via ozone itself is challenging, and the degradation process requires the activation of ozone molecules on the catalyst surface to generate free radicals for oxidative attack [25]. It is clear that the optimal activation performance for ozone is evidently facilitated by the synergistic effect of these two metal phases, resulting from a Ni/Mn ratio of 4:2.

The influence of Ni-Mn loading amounts over ACFA was also analyzed. When the ratio of Ni/Mn was fixed to 4:2, the loading amount of Ni-Mn is controlled at 8 wt.%, 20 wt.%, and 30 wt.% over ACFA, respectively, and the performance of obtained ACFA catalysts is shown in Figure 3b. The optimal ozone degradation performance of sodium acetate was obtained with the Ni-Mn loading amount of 20 wt.%. With the initial increase in Ni-Mn content, the catalyst will acquire a greater number of surface reactive sites, thereby enhancing ozone activation capability. However, this enhancement is rather limited, particularly when an excessive amount of metal oxide is loaded, as it may also impact pore structure and specific surface area, leading to a decline in catalyst performance. The synthesized fly ash catalyst shows a noticeable reduction in both specific surface area and pore structure when the Ni-Mn content is increased from 20 wt.% to 30 wt.%, as indicated in Table 1.

In order to further investigate the feasibility of fly ash as a catalyst, we also utilized γ-Al_2_O_3_ as a carrier to synthesize catalyst 0.2-Ni_0.67_Mn_0.33_OOH/γ-Al_2_O_3_ via the same method and compared its performance (Figure 3c) with catalyst 0.2-Ni_0.67_Mn_0.33_OOH/ACFA. Upon achieving adsorption stability, it was noted that the efficiency of sodium acetate degradation using the 0.2-Ni_0.67_Mn_0.33_OOH/γ-Al_2_O_3_ catalyst reached 49.3% after a reaction time of 60 min, which even exhibited lower efficiency than that of 0.2-Ni_0.67_Mn_0.33_OOH/ACFA. Firstly, it is evident that traditional carriers like γ-Al_2_O_3_ possess a large specific surface area, providing an ideal environment for direct pollutant adsorption and oxidation reactions. However, this advantage is not reflected in the case of sodium acetate degradation, indicating once again that ozone does not directly participate in its decomposition process. On the other hand, pre-treated fly ash without any loading still contains certain amounts of metal oxides such as iron [5], which also contribute to ozone activation for indirect oxidation of organics. A comparison between ACFA itself and parent γ-Al_2_O_3_ was also conducted to evaluate their respective catalytic performances. The sample of ACFA exhibits a certain level of ozone activation ability, which is manifested in its degradation performance on sodium acetate.

### 3.2. Morphology Characterization of x-Ni_y_Mn_1−y_OOH/ACFA Catalysts

**Figure 4:**
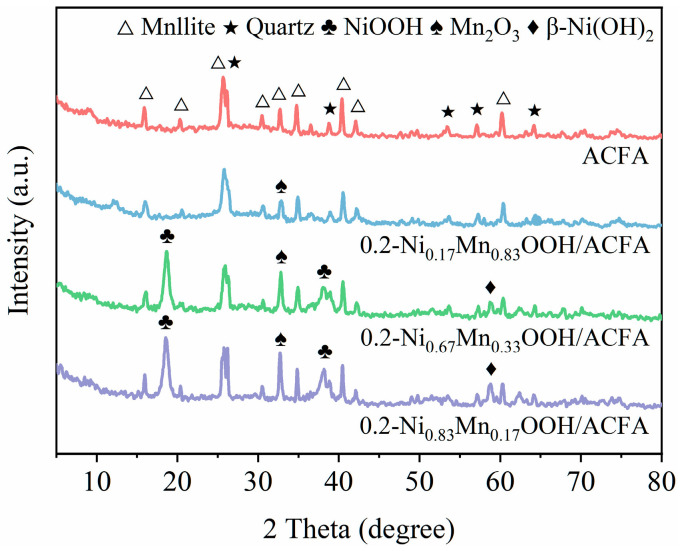
XRD spectra of pretreated fly ash and catalysts with different Ni/Mn ratios.

The XRD patterns of preprocessed fly ash and catalysts of 0.2-Ni_y_Mn_1−y_OOH/ACFA with different Ni/Mn ratios are shown in Figure 4. All samples exhibit the presence of mullite (2θ at 16.4°, 20.8°, 25.9°, 30.7°, 33.0°, 35.2°, 40.8°, 42.4° and 60.6°) and quartz (2θ at 26.1°, 39.2°, 53.5°, 56.8°, and 63.7°) with characteristic peaks [26], suggesting that no changes were made to the surface composition throughout the synthesis procedure. The absence of distinct NiOOH characteristic peaks for 0.2-Ni_0.17_Mn_0.83_OOH/ACFA can be attributed to the small amount of uniformly loaded NiOOH generated on the fly ash surface. With an increase amount of Ni(OH)_2_, significant diffraction peaks corresponding to NiOOH and Mn_2_O_3_ appear. The diffraction peak at 32.9° for Mn_2_O_3_ corresponds to the (222) crystal plane according to standard card JCPDS No.41-1442 [27]. The diffraction peaks at 18.3° and 37.8° correspond to typical crystalline phases of NiOOH on the (001) and (002) crystal planes [28]. Additionally, the diffraction peak at 59.0° also corresponds to β-Ni(OH)_2_ on the (110) plane [29].

**Figure 5:**
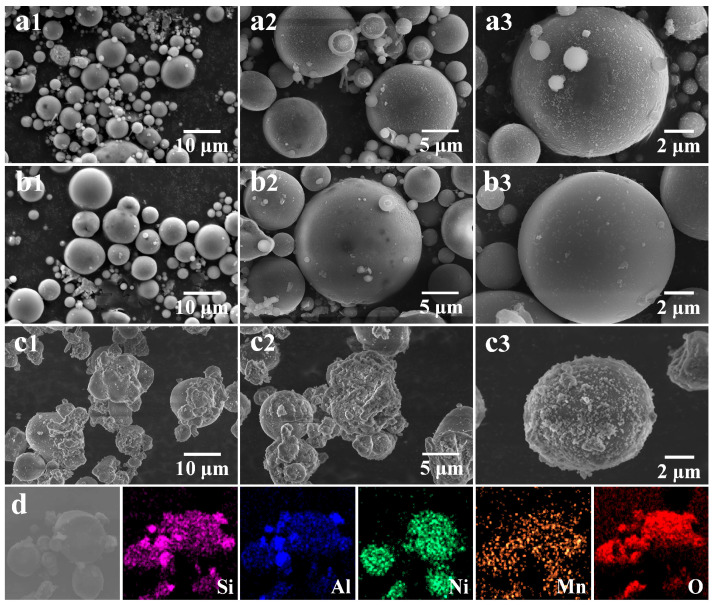
SEM image: (**a1**–**a3**) CFA, (**b1**–**b3**) ACFA, (**c1**–**c3**) 0.2-Ni_0.67_Mn_0.33_OOH/ACFA; (**d**) EDS-mapping of 0.2-Ni_0.67_Mn_0.33_OOH/ACFA.

**Figure 6:**
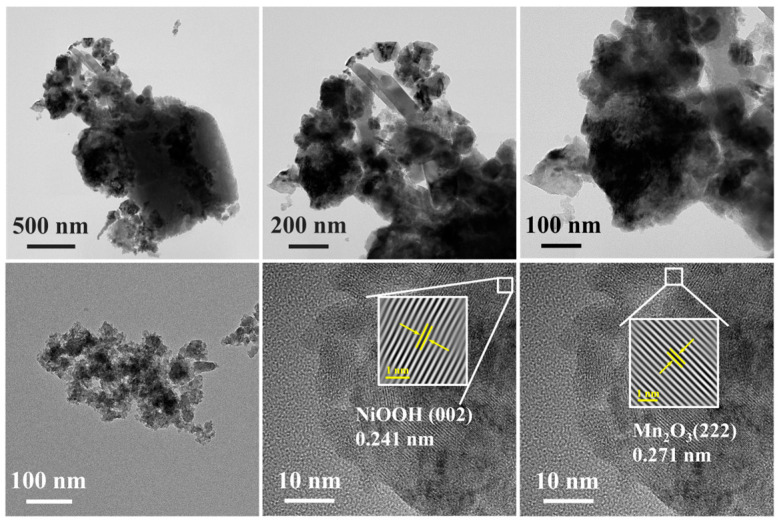
HR-TEM images of catalyst 0.2-Ni_0.67_Mn_0.33_OOH/ACFA.

The morphology of the acidified fly ash and catalyst 0.2-Ni_0.67_Mn_0.33_OOH/ACFA was characterized using SEM and TEM techniques as well. Based on the SEM image in Figure 5, it can be observed that the acidified fly ash showed a smoother surface structure than the original fly ash, but the external stable quartz phase did not change, which is mainly because the acid treatment can merely remove some impurities and alkali metals on the surface of fly ash. After the addition of Ni(OH)_2_, the surface of fly ash exhibits a more rugged and fragmented morphology, resulting in the formation of a well-defined pore structure and an increased specific surface area. The presence of Ni(OH)_2_ not only serves as a reactant in the redox reaction with KMnO_4_ on the surface of fly ash, resulting in the formation of NiOOH and Mn_2_O_3_, but its strong alkalinity also facilitates the dissolution of quartz and mullite structures on the fly ash surface via silicate reactions, leading to the development of a roughened morphology. EDS elemental analysis showed that Ni(OH)_2_ and KMnO_4_ reacted on the catalyst and loaded uniformly on the surface. It can be observed in Figure 5d that the distribution of elements Mn, Ni, and O is consistent with that of elements Al and Si, which indicates the formation of NiOOH and Mn_2_O_3_ over the surface of fly ash. The theoretical amounts of Ni and Mn are nearly consistent with the actual loadings of Ni and Mn on the fly ash surface (ICP test, Table 1), implying that a significant number of active phases comprising Ni and Mn eventually deposit onto the fly ash surface. For TEM images (Figure 6), the lattice spacing of 0.241 nm of 0.2-Ni_0.67_Mn_0.33_OOH/ACFA corresponds to the (002) crystal plane of NiOOH [28]. The measured lattice spacings of 0.271 nm for the (222) crystal plane of Mn_2_O_3_ [27].

### 3.3. Surface Properties of x-Ni_y_Mn_1−y_OOH/ACFA

**Figure 7:**
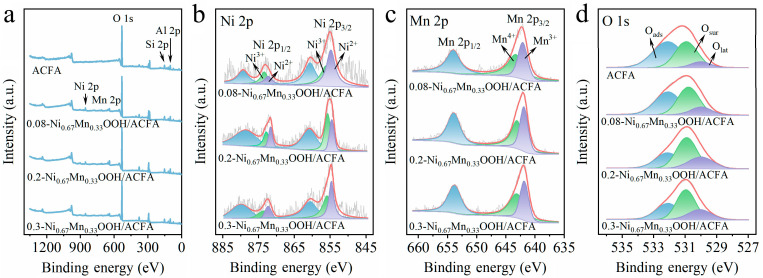
XPS spectra of pretreated fly ash and catalysts with different Ni/Mn ratios: (**a**) survey spectrum, (**b**) Ni 2p, (**c**) Mn 2p, (**d**) O 1s.

The chemical states of ACFA and different Ni_0.67_Mn_0.33_OOH/ACFA catalysts were analyzed using XPS. Figure 7a shows the full spectrum of catalyst loaded with Ni and Mn; the peaks at 855.4 eV and 642.4 eV represent Ni 2p and Mn 2p, respectively [30,31]. The Ni 2p spectrum exhibits four peaks with different binding energies, namely Ni 2p_3/2_, Ni 2p_1/2,_ and oscillating satellite peaks in Figure 7b. These reversed peaks correspond to Ni^2+^ at binding energies of 854.8 eV and 872.3 eV, as well as Ni^3+^ at binding energies of 856.0 eV and 873.5 eV [30,32]. In Figure 7c, the Mn 2p spectrum exhibits two peaks with different binding energies, namely the peaks of Mn 2p_3/2_ and Mn 2p_1/2_. The peak at 641.9 eV corresponds to Mn^3+^, while the peak at 643.1 eV corresponds to Mn^4+^ [31]. Figure 7d displays the XPS spectra of O 1s in catalysts with ACFA and varying Ni_0.67_Mn_0.33_OOH loads. The spectra of O 1s can be divided into three distinct peaks: those at around 530.0 eV are attributed to lattice oxygen (O_lat_) [33], those ranging from approximately 530.7 to 531.0 eV represent surface chemisorbed oxygen (O_sur_) (surface hydroxyl and oxygen vacancy (O_V_)) [34], and those between 532.0 and 532.1 eV correspond to surface adsorbed water molecules (O_ads_) [35].

**Figure 8:**
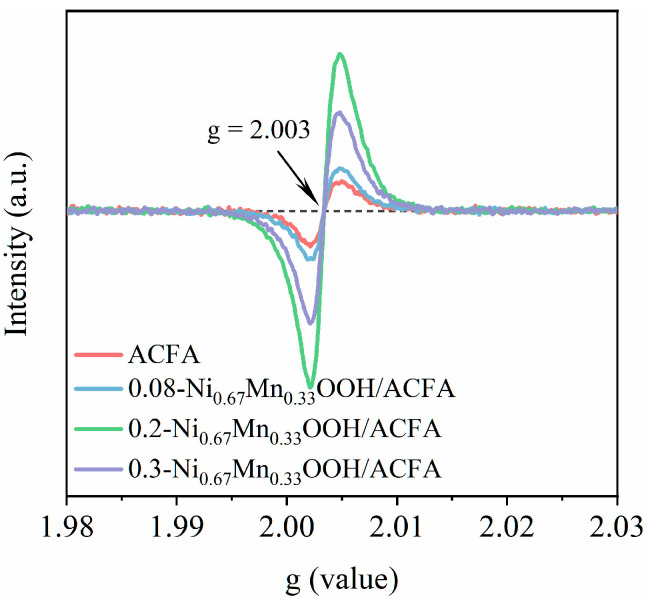
ESR spectra of pretreated fly ash and catalysts with different loading amounts of metal species.

As indicated in Table 2, with an increase in the loading of Ni_0.67_Mn_0.33_OOH from 8 wt.% to 20 wt.%, the ratio of Ni^2+^/Ni^3+^ decreases from 1.03 to 0.77, while Mn^3+^/Mn^4+^ and O_sur_ continue to rise. The reduction in high-valence Ni^3+^ to Ni^2+^ and the presence of reduced Ni^2+^ on the highly hydroxylated catalyst surface facilitate the formation of reactive species Ni-OH^+^ [19]. The electron cycling between valence changes in redox reactions involving Ni^2+^/Ni^3+^ and Mn^3+^/Mn^4+^ promotes ozone decomposition into reactive oxygen species. Additionally, an increase in Mn^3+^ also enhances the generation of oxygen vacancies [36], which serve as a platform for ozone adsorption and activation. EPR analysis (Figure 8) was also conducted on different samples, including ACFA and various loadings of Ni_0.67_Mn_0.33_OOH, revealing symmetric signals corresponding to oxygen vacancy electron paramagnetic resonance with g = 2.003 [37]. Notably, the intensity of oxygen vacancies observed in 0.2-Ni_0.67_Mn_0.33_OOH/ACFA was higher than that found in other samples, consistent with XPS analysis results regarding O_sur_ content, thus indicating that exposed oxygen vacancies provide more metal sites for ozone adsorption and conversion into reactive oxygen species, which is primarily responsible for sodium acetate oxidation by ozone. When increasing the loading amount of Ni_0.67_Mn_0.33_OOH from 20 wt.% to 30 wt.%, agglomeration occurs, resulting in a significant portion of the metal being difficult to expose and covered in the bulk phase. This not only leads to a decrease in specific surface area and stacking channel structure (see Table 1) but also leads to a gradual decrease in surface oxygen species and the formation of metal ions with higher valences and lattice oxygen content. From Table 2, it can be observed that the lattice oxygen content increases alongside an increase in the load amount from 20 wt.% to 30 wt.%.

### 3.4. Effect of Reaction Parameters on Catalytic Degradation Efficiency

**Figure 9:**
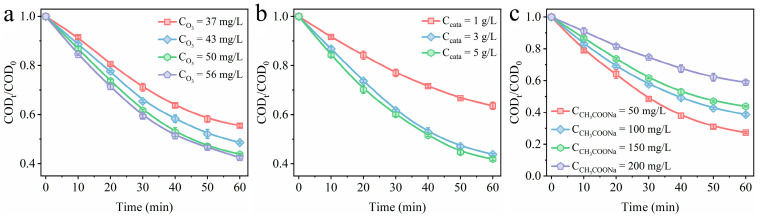
Exploring the influence of various reaction parameters on the efficiency of sodium acetate degradation using a 0.2-Ni_0.67_Mn_0.33_OOH/ACFA catalyst: (**a**) ozone concentration; (**b**) catalyst dosage; and (**c**) sodium acetate concentration (ozone concentration: 50 mg/L, catalyst dosage: 3 g/L, sodium acetate concentration: 150 mg/L, pH: 7.5, temperature: 25 °C).

The impact of ozone concentration, catalyst dosage, sodium acetate concentration, and pH value on the performance of catalyst 0.2-Ni_0.67_Mn_0.33_OOH/ACFA was investigated. As depicted in Figure 9a, the degradation efficiency of sodium acetate in the catalytic reaction system exhibited an upward trend with increasing ozone concentration. Specifically, after 60 min, the degradation efficiencies of sodium acetate were measured at 44.5%, 51.4%, and 56.2% for ozone concentrations of 37 mg/L, 43 mg/L, and 50 mg/L, respectively. However, as the ozone concentration was raised from 50 mg/L to 56 mg/L, only a marginal improvement up to a degradation rate of merely up to 57.5% was observed for sodium acetate. This can be attributed to the fact that elevating the ozone concentration within the reaction system generates more reactive oxygen species, which enhance sodium acetate degradation; however, due to limitations in catalyst dosage, excess ozone cannot be adequately decomposed and converted into reactive oxygen species. The impact of catalyst 0.2-Ni_0.67_Mn_0.33_OOH/ACFA dosage on the degradation of sodium acetate was also investigated. As shown in Figure 9b, there was a positive correlation between the catalyst dosage and the degradation efficiency of sodium acetate. With an increase in catalyst dosage from 1 g/L to 3 g/L, the rate of sodium acetate degradation rose from 36.4% to 56.2% after a reaction time of 60 min. This enhancement can be attributed to an augmented number of active sites resulting from higher catalyst amounts, which facilitates the adsorption process for both sodium acetate and ozone on the surface of the catalyst [38]. Further increase in the dosage of catalyst will not improve the degradation efficiency of sodium acetate, indicating that it reached a kinetic equilibrium state regarding ozone activation.

The end product of the oxidation process, sodium acetate, exhibits limited susceptibility to direct decomposition by ozone molecules [25]. Figure 9c illustrates the impact of varying sodium acetate concentrations on removal efficiency while keeping catalyst usage and ozone concentration constant. The COD values were 32.7 mg/L, 62.1 mg/L, 89.7 mg/L, and 121.1 mg/L at sodium acetate concentrations of 50 mg/L, 100 mg/L, 150 mg/L, and 200 mg/L, respectively. Specifically, the removal amount of sodium acetate consistently remains at ca. 40 mg/L when initial concentrations were set at 50 mg/L, 100 mg/L, 150 mg/L, and 200 mg/L, respectively. This observation substantiates the amount of sodium acetate removed is actually determined by the free radical content. The process described is an indirect oxidation method via free radical, wherein ozone does not undergo direct reaction with sodium acetate.

**Figure 10:**
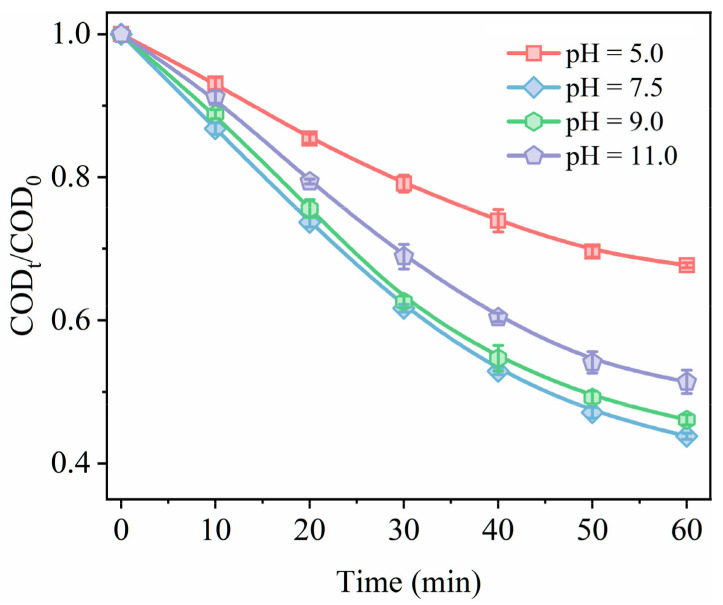
Effects of different pH values on the degradation performance of sodium acetate by using catalyst 0.2-Ni_0.67_Mn_0.33_OOH/ACFA (ozone concentration: 50 mg/L, catalyst dosage: 3 g/L, sodium acetate concentration: 150 mg/L, pH: 7.5, temperature: 25 °C).

The pH of the solution affects the decomposition of O_3_, changes the surface properties of the catalyst, and the adsorption of pollutants, which in turn affects the performance of catalytic ozonation [39]. Therefore, we also examined how pH affects the degradation of sodium acetate. Figure 10 illustrates the removal rate of sodium acetate for initial pH values ranging between 5.0, 7.5, 9.0, and 11.0. The degradation rate was found to be 32.3% when the initial pH was set at 5.0, indicating a significant inhibition in catalytic ozonation under acidic conditions. One crucial reason for this could be that low pH levels may disrupt catalyst stability, leading to catalyst leaching, which causes decreasing in active sites available for catalyzing ozonation [40]. Under neutral conditions, optimal catalytic oxidation of sodium acetate by ozone is consistently achieved. However, after a reaction time of 60 min at a pH value of 11.0, there was a slight decrease observed in sodium acetate degradation rate. This can be attributed to high OH^–^ concentrations leading to electrostatic repulsion between the catalyst and sodium acetate molecules, the surface of the catalyst coated with OH^–^ inhibited the contact of sodium acetate with the active site [41].

### 3.5. Study of Reaction Mechanism

**Figure 11:**
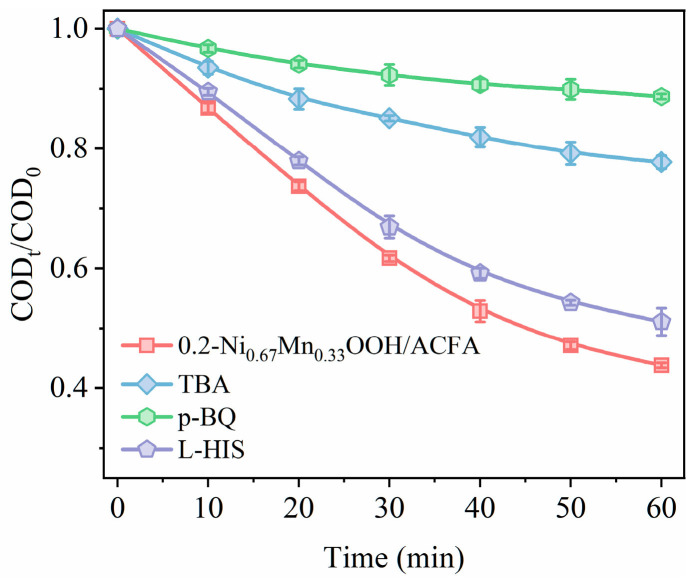
Effect of removal efficiency of sodium acetate over catalyst 0.2-Ni_0.67_Mn_0.33_OOH/ACFA with the addition of different quenchers. (ozone concentration: 50 mg/L, catalyst dosage: 3 g/L, sodium acetate concentration: 150 mg/L, pH: 7.5, temperature: 25 °C, TBA = 5 mmol/L, p-BQ = 0.5 mmol/L, L-HIS = 1 mmol/L).

A series of quenching experiments were conducted to determine the presence of active substances in the catalytic ozonation process using the 0.2-Ni_0.67_Mn_0.33_OOH/ACFA catalyst. As depicted in Figure 11, the impact of reactive oxygen species on the degradation performance of sodium acetate was assessed by quenching •O_2_^–^, •OH, and ^1^O_2_ with p-benzoquinone (p-BQ), tert-butanol (TBA), and L-histidine (L-HIS) as trapping agents, respectively [42,43]. The removal efficiency of sodium acetate decreased from 56.2% to 22.3% and 48.9% with the addition of TBA and L-HIS, respectively, while it dropped to 11.3% with the inclusion of p-BQ. This result suggests that •OH, •O_2_^–^, and ^1^O_2_ are active substances in the catalytic ozonation system employing the 0.2-Ni_0.67_Mn_0.33_OOH/ACFA catalyst, wherein •O_2_^–^ plays a predominant role.

**Figure 12:**
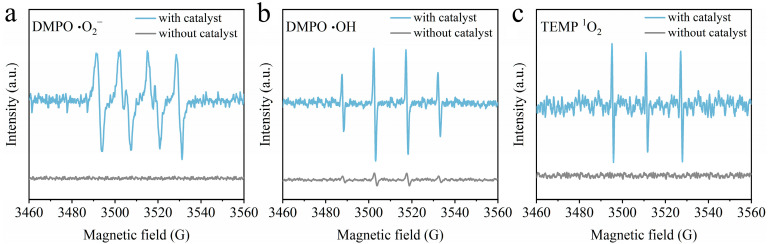
EPR spectra of 0.2-Ni_0.67_Mn_0.33_OOH/ACFA: (**a**) DMPO-•O_2_^–^, (**b**) DMPO-•OH, (**c**) TEMP-^1^O_2_.

EPR experiments were also conducted to further investigate the existence of •OH, •O_2_^–^, and ^1^O_2_ on the degradation of sodium acetate. 5,5-Dimethyl-1-pyrroline-N-oxide (DMPO) captured •O_2_^–^ and •OH to form 1:1:1:1 and 1:2:2:1 EPR signals [44], respectively. Additionally, 2,2,6,6-tetramethyl-4-piperidone hydrochloride (TEMP) captured ^1^O_2_ to produce a 1:1:1 EPR signal [45]. As shown in Figure 12, no characteristic signal was detected in the system without an added catalyst. However, after adding catalyst 0.2-Ni_0.67_Mn_0.33_OOH/ACFA, corresponding characteristic signal peaks were observed. This indicates that in the catalytic ozonation system, ozone is activated by metal sites and oxygen vacancies on the catalyst surface to generate reactive oxygen species. The statement suggests that in the catalytic ozonation system, ozone is activated on the catalyst surface, leading to the generation of reactive oxygen species.

**Figure 13:**
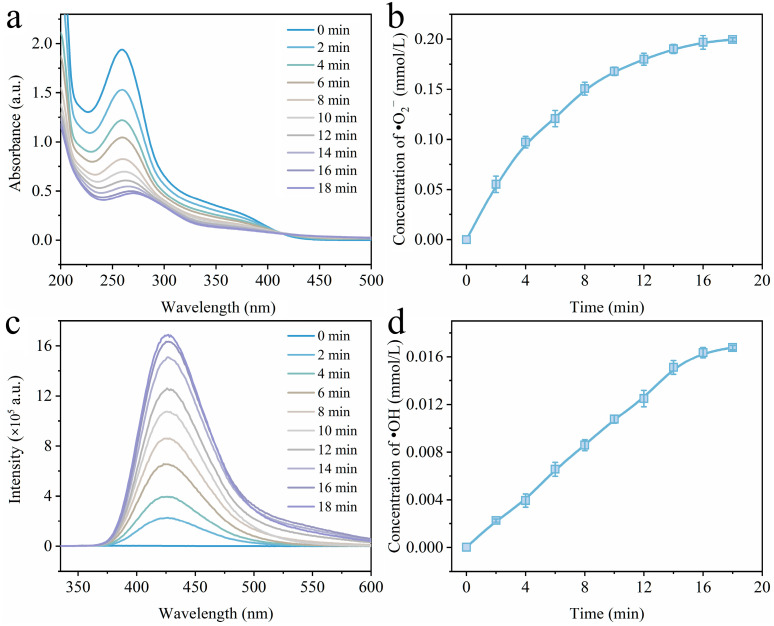
(**a**) UV profile of NBT solution; (**b**) NBT absorption spectroscopy to determine the concentration of •O_2_^–^; (**c**) fluorescence profile of TAOH solution; (**d**) Photoluminescence spectroscopy of PL to determine the concentration of •OH.

The •O_2_^–^ and •OH that affect the sodium acetate degradation over catalyst 0.2-Ni_0.67_Mn_0.33_OOH/ACFA were further analyzed via quantitative experiments. Since nitroblue tetrazolium chloride (NBT) can capture •O_2_^–^, it is quantified using the NBT method [46]. As shown in Figure 13a, the absorbance at 259 nm gradually decreased with increasing reaction time, indicating continuous generation of •O_2_^–^ during catalytic ozone oxidation. The concentration of •O_2_^–^ at 18 min was determined to be 0.199 mmol/L (Figure 13b). Terephthalic acid (TA) effectively traps •OH and produces a highly fluorescent product of 2-hydroxyterephthalic acid (TAOH) at 425 nm [47], as demonstrated in Figure 13c. The intensity of the fluorescence peak significantly increased with prolonged catalytic ozonization time. The intensity of •OH was calculated based on the fluorescence peak intensity (Figure 13d), reaching a concentration of 0.016 mmol/L at 18 min, which is much lower than that of generated •O_2_^–^. This finding is consistent with the results from quenching experiments shown in Figure 11, suggesting that •O_2_^–^ plays a dominant role in catalyzing ozone degradation for sodium acetate.

Based on the aforementioned experimental results, the mechanism of catalytic ozone degradation of sodium acetate can be deduced as follows. The Mn^3+^ acts as an active site or electron donor in the reaction, undergoing electron loss and oxidation to Mn^4+^, while Ni^3+^ is reduced to Ni^2+^. Ozone molecules are adsorbed onto the vacant surface of Mn^3+^ and further activated into •O_2_^–^ and •OH for sodium acetate degradation. Due to the high concentration of formed •O_2_^–^, a portion of it can also generate ^1^O_2_, which has been reported as a secondary free radical with significant oxidation properties [48]. In order to maintain electrostatic equilibrium on the catalyst, a redox reaction will also occur between Ni^2+^ and Mn^4+^, resulting in the regeneration of Ni^3+^ and Mn^3+^, thus achieving cycling between Ni^2+^/Ni^3+^ and Mn^3+^/Mn^4+^ [49].

Mn^3+^ + O_3_ + H_2_O → Mn^4+^ + •O_2_^−^ + •HO_3_ (1)

Ni^3+^ + OH^–^ + •HO_3_ → Ni^2+^ + •OH + O_2_ (2)

•HO_3_ → O_2_ + •OH (3)

•OH + •O_2_^–^ → ^1^O_2_ + OH^–^ (4)

Mn^4+^ + Ni^2+^ → Mn^3+^ + Ni^3+^ (5)

## 4. Conclusions

In this work, we have created a catalyst for ozone support by employing fly ash as a carrier and integrating MnO_x_/NiOOH as the active phase. NiOOH and Mn_2_O_3_ are produced via a one-step reaction of Ni(OH)_2_ and KMnO_4_, eliminating the need for high-temperature calcination of synthetic metal oxides, which makes the reaction more environmentally friendly. It shows good ozone oxidation performance, and the removal efficiency of sodium acetate can reach 57.5% under the reaction condition of ozone concentration at 50 mg/L, catalyst dosage at 3 g/L, sodium acetate concentration at 150 mg/L, and pH at 7.5. By loading metal species on the surface, more oxygen vacancy defects are created on the surface of the fly ash, which thus convert the absorbed O_3_ into •O_2_^–^, •OH, and ^1^O_2_. The quantitative analysis of free radicals also indicates that •O_2_^–^ is the largest contributor to the oxidation of sodium acetate. Combined with the analysis of XPS, the reasonable reaction mechanism over this catalyst surface was deduced. Ozone molecules are adsorbed onto the vacant surface of Mn^3+^ and further activated into •O_2_^–^ and •OH for sodium acetate degradation. And a redox reaction will also occur between Ni^2+^ and Mn^4+^, resulting in the regeneration of Ni^3+^ and Mn^3+^, thus achieving cycling between Ni^2+^/Ni^3+^ and Mn^3+^/Mn^4+^. This work proves that fly ash, as a kind of solid waste, is possible to purify industrial wastewater by a simple synthesis method and low cost.

## Figures and Tables

**Table 1:** Specific surface area, pore size, and ICP elemental tests of pretreated fly ash and catalysts with different Ni/Mn ratios.

Samples	S_BET_ (m^2^/g)	V_total_ (cm^3^/g)	Pore Size (nm)	ICP Test of Catalyst Elements	
				Ni wt.%	Mn wt.%
CFA	1.47	0.0047	8.14	\	\
ACFA	11.37	0.0083	9.72	\	\
0.08-Ni_0.67_Mn_0.33_OOH/ACFA	34.22	0.12	10.16	5.11	2.43
0.2-Ni_0.67_Mn_0.33_OOH/ACFA	22.83	0.089	10.38	12.78	5.41
0.3-Ni_0.67_Mn_0.33_OOH/ACFA	14.28	0.051	8.14	18.37	8.24
γ-Al_2_O_3_	164.66	0.57	6.23	\	\
0.2-Ni_0.67_Mn_0.33_OOH/γ-Al_2_O_3_	122.49	0.38	5.71	\	\

**Table 2:** XPS analysis of pretreated fly ash and catalysts with different Ni/Mn ratios.

Samples	XPS Analysis				
	Ni^2+^/Ni^3+^	Mn^3+^/Mn^4+^	O_ads_	O_sur_	O_lat_
ACFA	–	–	0.54	0.38	0.08
0.08-Ni_0.67_Mn_0.33_OOH/ACFA	1.03	0.80	0.44	0.42	0.14
0.2-Ni_0.67_Mn_0.33_OOH/ACFA	0.77	0.95	0.31	0.48	0.21
0.3-Ni_0.67_Mn_0.33_OOH/ACFA	0.86	0.74	0.32	0.45	0.23

## Data Availability

Data are contained within the article. The data presented in this study are available upon request from the corresponding author.
